# Labeling mesenchymal cells with DMSA-coated gold and iron oxide nanoparticles: assessment of biocompatibility and potential applications

**DOI:** 10.1186/s12951-016-0213-x

**Published:** 2016-07-18

**Authors:** Luisa H. A. Silva, Jaqueline R. da Silva, Guilherme A. Ferreira, Renata C. Silva, Emilia C. D. Lima, Ricardo B. Azevedo, Daniela M. Oliveira

**Affiliations:** IB-Departamento de Genética e Morfologia, Universidade de Brasília-UNB, Campus Universitário Darcy Ribeiro-Asa Norte, Brasília, DF CEP 70910-970 Brazil; Instituto de Química, Universidade Federal de Goias, Goiânia, GO Brazil; Instituto Nacional de Metrologia, Rio de Janeiro, RJ Brazil

**Keywords:** Mesenchymal stem cells, Iron oxide nanoparticle, Gold nanoparticles, Biocompatibility, Computed microtomography, Magnetic targeting, DMSA-nanoparticles

## Abstract

**Background:**

Nanoparticles’ unique features have been highly explored in cellular therapies. However, nanoparticles can be cytotoxic. The cytotoxicity can be overcome by coating the nanoparticles with an appropriated surface modification. Nanoparticle coating influences biocompatibility between nanoparticles and cells and may affect some cell properties. Here, we evaluated the biocompatibility of gold and maghemite nanoparticles functionalized with 2,3-dimercaptosuccinic acid (DMSA), Au-DMSA and γ-Fe_2_O_3_-DMSA respectively, with human mesenchymal stem cells. Also, we tested these nanoparticles as tracers for mesenchymal stem cells in vivo tracking by computed tomography and as agents for mesenchymal stem cells magnetic targeting.

**Results:**

Significant cell death was not observed in MTT, Trypan Blue and light microscopy analyses. However, ultra-structural alterations as swollen and degenerated mitochondria, high amounts of myelin figures and structures similar to apoptotic bodies were detected in some mesenchymal stem cells. Au-DMSA and γ-Fe_2_O_3_-DMSA labeling did not affect mesenchymal stem cells adipogenesis and osteogenesis differentiation, proliferation rates or lymphocyte suppression capability. The uptake measurements indicated that both inorganic nanoparticles were well uptaken by mesenchymal stem cells. However, Au-DMSA could not be detected in microtomograph after being incorporated by mesenchymal stem cells. γ-Fe_2_O_3_-DMSA labeled cells were magnetically responsive in vitro and after infused in vivo in an experimental model of lung silicosis.

**Conclusion:**

In terms of biocompatibility, the use of γ-Fe_2_O_3_-DMSA and Au-DMSA as tracers for mesenchymal stem cells was assured. However, Au-DMSA shown to be not suitable for visualization and tracking of these cells in vivo by standard computed microtomography. Otherwise, γ-Fe_2_O_3_-DMSA shows to be a promising agent for mesenchymal stem cells magnetic targeting.

## Background

Due to the progression of nanotechnology, there are new materials at the nanometer scale that have been introduced to the Medicine [1]. One promising medical application of nanomaterials is the use of inorganic nanoparticles within mesenchymal stem cells (MSCs)-based therapies: nanomaterials have facilitated not only the investigation of stem cells’ biology but also the development of new approaches for their expansion, differentiation and transplantation. Some of these nanomaterials possess chemical, optical or magnetic properties which can be used for visualization and tracking of MSCs [2–6].

Among the nanoparticles, iron oxide nanoparticles (IONPs) have great prominence because of their superparamagnetism, a property highly valued for biomedical applications [7]. Due to the strong signal that IONPs generate in magnetic resonance imaging, it is possible to visualize cells in microscopic levels and get specific information about their distribution in vivo [3, 7–9]. In addition, IONPs’ magnetic properties can be explored for magnetically assisted cell delivery and retention in target organs [10–12], which is one of major current challenges in cell therapy. Lastly, IONPs superparamagnetism has also been applied in hyperthermia therapies in tumors, taking advantage of MSCs tropism to tumor cells [9].

Recently, another class of inorganic nanoparticles, gold nanoparticles (Au-NPs), has been explored in cell based therapies. It is known that nanoscale gold strongly absorbs and scatters visible light—a phenomenon that is based on the occurrence of surface plasmons [13]. Therefore, Au-NPs have been used for stem cells marking, which can be detected in vivo by fluorescence or photothermal imaging [6, 14–16]. Moreover, as Au-NPs scatter X-rays efficiently, labeled MSCs can also be detected by computed tomography, a technique that provides greater spatial resolution compared to magnetic resonance imaging [5, 6, 17, 18].

Despite their potential in clinical practice, the impacts of IONPs and Au-NPs on MSCs are not entirely clear. First, both materials can be toxic to cells. It is known that transition metals such as iron, when retained in excess in cell cytoplasm in a non-complexed form, act as catalysts for oxidation reactions of biomolecules, then increasing the rate of free radicals generation [19, 20]. On the other hand, even though macroscopic gold appears chemically inert, at the nanometric scale it may induce oxidative stress and it can bind permanently to nuclear and mitochondrial DNA [21–26]. There are also some studies showing unexpected physiological alterations in nanoparticle labeled MSCs, such as changes in differentiation ability [25–29] and in growth rates [26, 30]. These observations reinforce the importance of conducting previous biocompatibility tests of these nanoparticles before in vivo procedures [31, 32].

Coating nanoparticles with a proper surface modification is a strategy largely used to decrease potential toxic effect on cells. Biomedical applications of nanoparticles require surface modifications of nanoparticles in order to make them non-toxic, biocompatible, non-agregable and stable [33]. Surface functionalization of inorganic particles with 2, 3-dimercaptosuccinic acid (DMSA) is considered to be a promising strategy to increase biocompatibility [34, 35].

Here, we aimed to investigate the biocompatibility and potential use of two inorganic nanoparticles coated with DMSA, iron oxide nanoparticles coated with DMSA (γ-Fe_2_O_3_-DMSA) and gold nanoparticles coated with DMSA (Au-DMSA) used to label human MSC. Further, these nanoparticles were tested on two practical applications: Au-DMSA nanoparticles were tested as tracers for MSC in vivo tracking by computed tomography; and γ-Fe_2_O_3_-DMSA as agents for magnetic targeting of MSC.

## Methods

### Nanoparticles synthesis and characterization

Maghemite nanoparticles were prepared via oxidation of precursor magnetite nanoparticles, as described in the literature. γ-Fe_2_O_3_ nanoparticles were synthesized by mixing ferric and ferrous chloride aqueous solutions (2:1 molar ratio) with concentrated ammonia aqueous solution followed by vigorous stirring. The black magnetite precipitate was washed several times with water and collected by a magnet. The oxidation of magnetite to maghemite was carried out by refluxing the nanoparticles in 0.5 mol/L hydrochloric acid (HCl) solution under oxygen flux at 96 °C, yielding a brownish colloidal suspension. The brown precipitate was extensively washed using the 1 mol/L HCl solution and decanted by a magnet. The sample was redispersed in water and dialyzed against demineralized water to produce an aqueous acidic magnetic dispersion. The meso-2,3-dimercaptosuccinic acid (DMSA) coated maghemite nanoparticles (γ-Fe_2_O_3_-DMSA) was prepared according the early described protocol [36]. Five millilitre of DMSA (Acros Chemicals) stock solution (0.3 mol/L) were added to the 25 mL of the dispersion of maghemite nanoparticles in a molar ratio DMSA/Fe of 11 %. The dispersion was shacked for 12 h at room-temperature. Then, the dispersion was dialyzed for 12 h against demineralized water to eliminate the free DMSA out from the bulk solution. The pH was adjusted to the range of 7.0–7.2 and the suspension containing the maghemite nanoparticles functionalized with DMSA was purified against large aggregates by centrifugation at 5000 rpm for 10 min.

The total iron and gold content in the suspensions were determined by Atomic absorption spectrophotometry in a commercial Perkin-Elmer 5000 system (Perkin-Elmer, Norwalk, USA). The Fe^2+^/Fe^3+^ ratio was determined by the 1–10 phenanthroline colorimetric method. X-ray powder diffraction (XRD) data were collected by a XRD-6000 diffractometer (Shimadzu, Kyoto, Japan). The average diameter of the nanocrystalline domain (*d*) was estimated using the Scherrer’s equation [37]. Electronic micrographs of maghemite nanoparticles were obtained with a JEOL JEM 2100 Transmission Electron Microscopy (TEM). Hydrodynamic diameter and zeta potential measurements were performed using the Malvern Zetasizer Nano-ZS (Malvern Instruments Ltd., Worcestershire, UK).

The synthesis of the DMSA coated gold nanoparticles (Au-DMSA) was carried out by following the method purposed by Gao et al. [38]. Shortly, 5 mL of an aqueous DMSA solution 1.8 × 10^−3^ mol/L was added to 25 mL of an aqueous HAuCl_4_ solution 6 × 10^−4^ mol/L at the boiling point, and the system was maintained under stirring by 15 min. After cooling to room temperature the colloidal suspension was against demineralized water and stored in the dark.

The total gold content in the metallic suspensions were determined by Atomic absorption spectrophotometry in a commercial Perkin-Elmer 5000 system (Perkin-Elmer, Norwalk, USA). Au-DMSA nanoparticle morphology was examined using a JEOL JEM 2100 TEM. Au-DMSA particle size was measured by dynamic light scattering (DLS) and zeta potential measurements were performed using Malvern Zetasizer Nano-ZS.

### Cells

Dental pulp tissues were obtained from the permanent teeth of patients (17–43 years of age) under approval of the Ethical Committee of Health Sciences Faculty of the University of Brasília (Brazil) (Project number 023/08), as previously described [39]. All pulp tissues were washed with a-MEM, digested with 3 mg/mL collagenase type I (Gibco) in supplemented medium for 60 min at 37 °C. After enzymatic digestion, cell suspension was washed three times by centrifugation (10 min at 750*g*) in culture medium and placed into 6-well plates. The human MSC obtained were cultured in Low-Glucose Dulbecco’s Modified Eagle Medium (DMEM-LG) (GIBCO^®^, Invitrogen,Carlsbad, CA) supplemented with 1 % l-glutamine (GIBCO^®^, Invitrogen, Carlsbad, CA), 1 % antibiotic–antimycotic (10,000 UI/mL penicillin, 10,000 mg/mL streptomycin and 25 µg/mL Amphotericin B) (GIBCO^®^, Invitrogen, Carlsbad, CA) and 10 % fetal bovine serum. MSC were grown under standard cell culture conditions (37 °C, 5 % CO_2_) and have been maintained to their confluence below 80 %. Only passage 3–4 cells were used in this study.

### Assessment of cell viability and morphology

The MSC were exposed to γ-Fe_2_O_3_-DMSA (15, 30, 60 and 80 µg iron/mL) and to Au-DMSA (52, 90 and 130 µg gold/mL) in growth medium for 02, 06 or 24 h. After each exposition time, labeled MSC viability was evaluated by MTT (3-[4,5-dimethylthiazol-2-yl]-2,5-diphenyltetrazolium bromide) assay [40], by Trypan blue exclusion assay [41] and by cell morphology analysis. For MTT assay, MSC were exposed to γ-Fe_2_O_3_-DMSA and Au-DMSA during 02, 06 or 24 h. In Trypan blue exclusion assay, MSC were incubated with nanoparticles only during 24 h. For evaluation of cell morphology, labeled MSC were incubated for 24 h with DMEM-LG with serum (negative control group), with DMEM-LG without serum (positive control group), with γ-Fe_2_O_3_-DMSA nanoparticles (80 µg iron/mL) or with Au-DMSA nanoparticles (90 µg gold/mL). After incubation time, the cells were stained with Instant Prov Kit (Newprov) according to the manufacturer’s recommendations and observed analyzed using an inverted microscope (Axiovert 100, Zeiss).

### Nanoparticle uptake

After 24 h of incubation, the cells were washed, ressuspended in PBS and counted using an automatic counter (Scepter ™, Millipore). Prussian blue staining was used to quantify the amount of uptaken γ-Fe_2_O_3_-DMSA, as described by Boutry et al. [42], with some modifications: 100 µl of 5 N HCl were added to the samples, incubating them at 80 ℃ for 4 h to lyse cells. After that, the samples were transferred to a 96-well polystyrene plate and 100 mL of 5 % potassium ferrocyanide were added. The absorbance of samples at 630 nm was measured and data were compared with a standard curve whose function relates the Prussian blue OD_630 nm_ with iron concentration in the sample. The results are expressed as iron per cell.

Inductively coupled plasma optical emission spectrometry (ICP-OES) technique (Spectro Arcos, Ametek) was used to measure the amount of intracellular gold after exposure to the Au-DMSA (90 µg gold/mL) for 24 h.

### Transmission electron microscopy analysis

After incubation of MSC with Au-DMSA (90 µg gold/mL) and γ-Fe_2_O_3_-DMSA (80 µg iron/mL), as described previously, they were fixed in modified Karnovsky’s fixative (2 % paraformaldehyde, 2.5 % glutaraldehyde in 0.1 M sodium cacodylate buffer, pH 7.2) for 2 h at room temperature. Samples were postfixed in solution containing 1 % osmium tetroxide, 0.8 % potassium ferricyanide, and 5 mM calcium chloride and contrasted in bloc with 0,5 % uranyl acetate. Samples were then dehydrated in acetone and embedded in Spurr. Semi-thin sections (3 μm) were stained with toluidine blue and examined under a light microscope to localize cells with visible nucleus. Ultra-thin sections (70 nm) were examined using a Tecnai Spirit G2 TEM (FEI, USA).

### MSC differentiation

To verify if both nanoparticles interfere with the MSC ability to differentiate, the cells were cultured in medium enriched with inducing agents, after their exposure to γ-Fe_2_O_3_-DMSA (80 µg/mL) and Au-DMSA (90 µg/mL) for 24 h. For the experiments of differentiation into osteoblasts, the cell inducers used were dexamethasone (5 × 10^−6^ M); ascorbic acid (2.8 × 10^−4^ M); and β-glycerol phosphate (10^−2^ M). Subsequently, cytochemical analyses with specific labeling with dyes were performed. The MSCs differentiated into osteoblasts were fixed in 50 % ethanol for 15 min at 4 °C and stained with a solution of Alizarin Red S 1 %. Quantitative analysis of osteogenic differentiation was performed by quantification of Alizarin Red S adhered to calcified tissues, following the protocol adopted by Gregory et al. [43], and measurement of alkaline phosphatase (ALP) activity, by the colorimetric method of para- nitrophenol [44], using the kit SIGMAFAST* p*-Nitrophenyl phosphate tablets (Sigma) according to the manufacturer’s recommendations. The corresponding values of enzyme activity in milliunits (mIU) per milliliter were divided by the total protein content (in µg) of the monolayer, estimated by the Lowry method [45].

To induce differentiation into adipocytes, the inducers used were dexamethasone (5 × 10^−6^ M); 0.3-isobutyl-methylxanthine (4.5 × 10^−4^ M); insulin (5 μg/mL); and indomethacin (3 × 10^−4^ M). After 24 days, the cells differentiated into adipocytes were fixed with formaldehyde solution for 15 min, and stained with a solution of “Oil Red O” at 0.3 % for 20 min for cytochemical analysis. An indirect measurement of adipogenesis was performed by quantification of Oil Red O in MSC monolayers adding 100 % isopropanol to extract the dye and measure its absorbance at 510 nm.

### MSC growth curve

MSC were cultivated in 75 cm^2^ culture flasks until they reached 80 % confluence. The growth curve was performed by incubating the cells with DMEM-LG (control), with γ-Fe_2_O_3_-DMSA (80 µg/mL), and with Au-DMSA (90 µg/mL). The cells were washed, disassociated from the flasks and seeded in 12-well cell culture plates at a starting concentration of 10,000 cells per well. Then, they were collected after six different times of incubation (2, 4, 6, 8, and 10 days), stained with Trypan blue dye and counted. The results are expressed as percentage of live cells.

### Suppression of lymphocyte proliferation by MSC

Human mononuclear cells were isolated by Ficoll-Paque gradient. Briefly, blood were collected, stored in tubes containing anticoagulants, diluted in phosphate buffered saline (PBS) and transferred to a centrifuge tube, over a Ficoll-Paque layer (ρ = 1.077 g/mL). The tubes were centrifuged at 2000 rpm for 25 min, and the mononuclear cell layer obtained was then transferred to a new centrifuge tube. The mononuclear cells were counted using a Neubauer chamber (Gibco), resuspended at a final concentration of 10^7^ cells/mL and labeled with Carboxyfluorescein succinimidyl ester (CFSE), according to manufacturer recommendations (CellTrace™ CFSE dye, Life Technologies). Lymphocytes then received allogeneic stimulus in the presence or absence of MSC for 5 days.

### Murine model of silicosis

All experimental protocols with animals in this study were approved by the animal experimentation ethics committee of University of Brasilia (certificate # 99769/2012). C57BL/6 mice, 8 weeks old, were randomly divided into: control group, instilled intratracheally with 50 μL of sterile saline; and silicosis group, instilled intratracheally with a silica particle suspension (20 mg/50 μL of saline). In this model, the pathophysiological characteristics of silicosis are observed 15 days after installation of crystalline silica [46].

### Computed tomography analysis

The application of Micro-CT to tracking Au-DMSA labeled MSCs in vivo was tested using a 1076 Skyscan microtomography device (Skyscan, Aartselaar, Belgium). The mice were analyzed daily in the equipment until the 7th day after the inoculation of Au-DMSA labeled MSCs or saline. Images were acquired using voltage 50 kV, current 180 mA, 0.5 mm aluminum filter and isotropic voxel size of 18 μm. For the two-dimensional image reconstruction, we used NRecon software (V 1.6.9, 64 bit version with GPU acceleration, Skyscan, Kontich, Belgium). For three-dimensional reconstructions, we used CTVox software (V 1.5.0, 64 bit version, Skyscan, Kontich, Belgium) and CTVol software (2.2 V, 64 bit version, Skyscan, Kontich, Belgium). Analyses of reconstructions were performed using the software CTAnalyzer (V 1.5.0, 64 bit version, Skyscan, Kontich, Belgium). The most appropriate parameters of smoothing, ring artifacts correction and beam-hardening correction were used. All acquisition and reconstruction parameters were the same for all mice.

### Magnetic targeting of γ-Fe_2_O_3_-DMSA labeled MSC

γ-Fe_2_O_3_-DMSA nanoparticles were tested as potential agents for MSC magnetic targeting to injured lungs. The cells were incubated with 80 µg/mL of γ-Fe_2_O_3_-DMSA for 24 h and inoculated into silicotic mice. Neodymium circular magnets (20 mm in diameter and 2 mm height) were held in the thoracic region of some animals for up to 24 h. 48 h after inoculation of MSC, the animals were euthanized and their lungs were collected for iron quantification following the protocol described by Boutry et al. [42], and histological analysis. Slides containing lung sections were also stained with Prussian blue.

### Statistical analysis

The normality of the data was analyzed by the Kolmogorov–Smirnov test. Then, the parametric test ANOVA, followed by post hoc Tukey’s test was performed for statistical comparison of data of the following experiments: Differences were considered statistically significant with p < 0.05.

## Results

### Fe-DMSA and Au-DMSA characterization

**Fig. 1:**
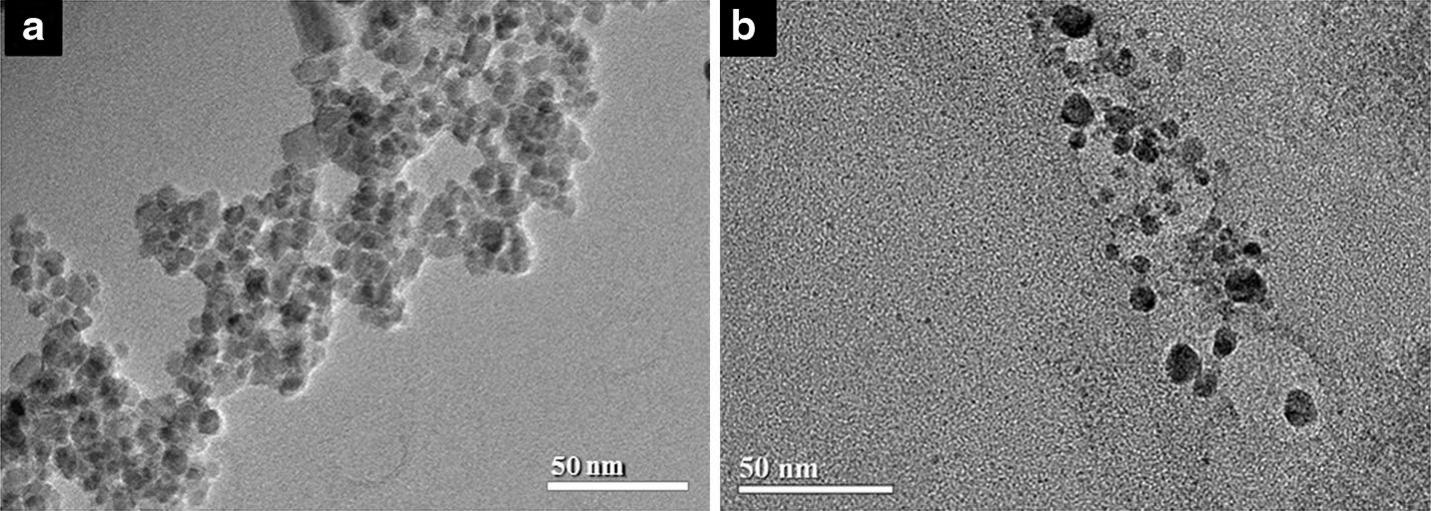
Transmission electron microscopy analysis of γ-Fe_2_O_3_-DMSA and Au-DMSA. **a** TEM micrographs of Fe-DMSA nanoparticles, *bars* 50 nm. **b** TEM micrographs of Au-DMSA nanoparticles, *bars* 50 nm

According to Atomic absorption spectrophotometry data, the γ-Fe_2_O_3_-DMSA solution used in this study has 4.09 mg of iron per mL. In addition, γ-Fe_2_O_3_-DMSA nanoparticles present zeta potential of −43 ± 0.66 mV, irregular shape from square to sphere in TEM micrographs (Fig. 1a) and nanocrystalline diameter in a range of 5–18 nm, as determined by Scherrer’s equation with XRD data (data not showed).

On the other hand, Au-DMSA solution presented low gold concentration—0.07 mg/mL—hindering XRD analysis; thus, Au-DMSA particle size was determined by Dynamic Light Scattering: 26.4 ± 0.96 nm. Similarly to γ-Fe_2_O_3_-DMSA nanoparticles, Au-DMSA also has negative zeta potential—40.8 ± 3.70 mV—and has spherical morphology in TEM micrographs (Fig. 1b).

### Cytotoxicity assays

**Fig. 2:**
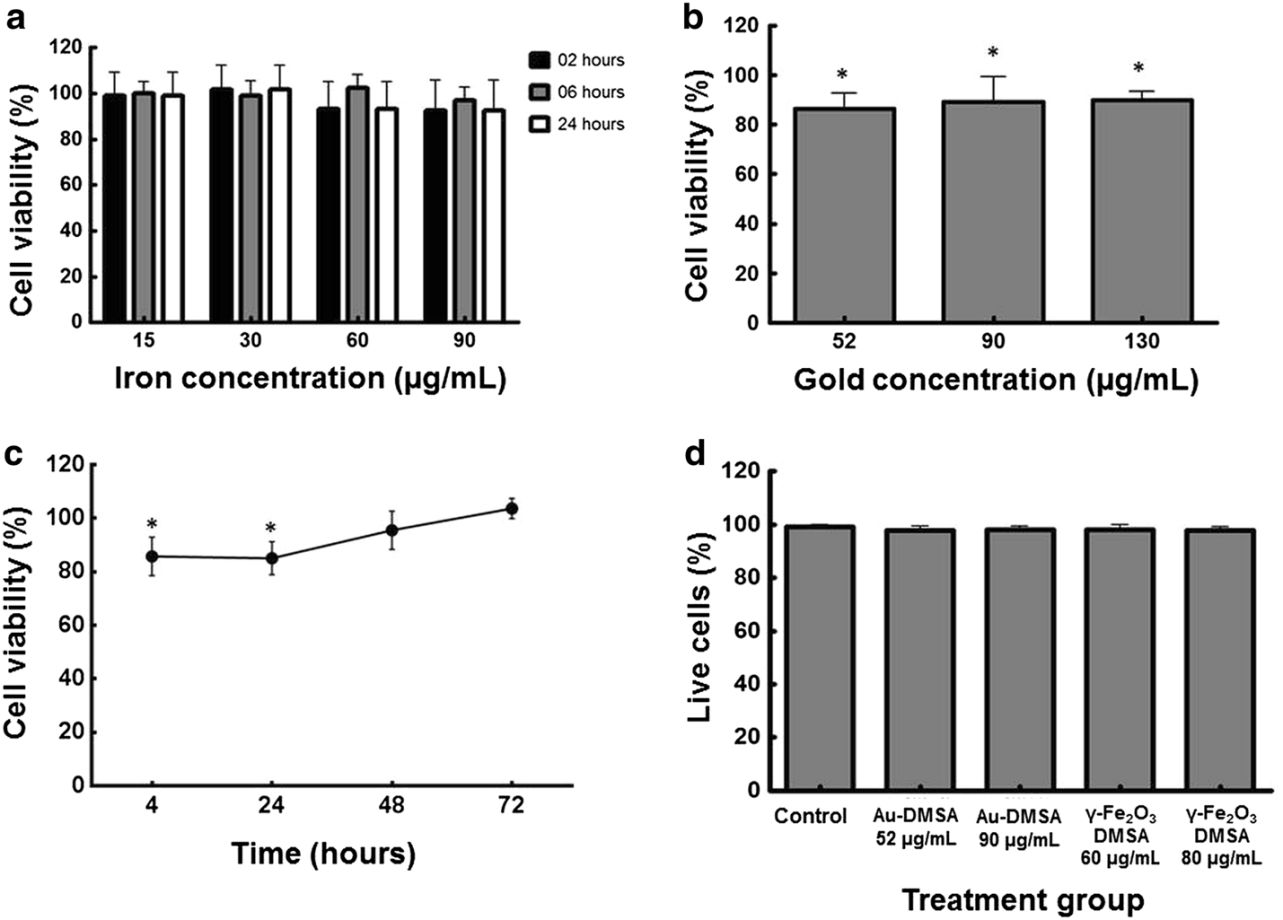
Cell viability assessment by MTT and Trypan-blue staining. **a** MTT assay of γ-Fe_2_O_3_-DMSA labeled MSC. The data express average percentage and standard deviation of MSC that have remained viable after exposure to γ-Fe_2_O_3_-DMSA in four different concentrations at three different exposure times. **b** MTT assay of Au-DMSA labeled MSC. The average percentage and standard deviation of viable MSC are represented after 24 h of exposure to three different concentrations of Au-DMSA. (*) Significant reduction between the cells of the three experimental groups compared to the control group (p < 0.01). **c** MTT assay of Au-DMSA labeled MSC 4, 24, 48 and 72 h after incubation with the nanoparticles (90 µg/mL) during 24 h. There were significant differences between control cells and labeled cells only 4 and 24 h after exposure (p < 0.05). **d** Cell viability test by trypan-blue staining. The data express the mean percentage and standard deviation of MSC that remained alive after 24 h of exposure to 52 and 90 µg/mL of Au-DMSA and 60 and 80 µg/mL of γ-Fe_2_O_3_-DMSA

According to the MTT assay, MSC exposed to γ-Fe_2_O_3_-DMSA nanoparticles (15, 30, 60 and 80 µg iron/mL) remained viable, with no difference between experimental groups and their respective control groups at any incubation time (Fig. 2a). Differently, there was a 20–25 % reduction in the cell viability when they were exposed to Au-DMSA for 24 h, compared to the control cells (Fig. 2b). However, no difference was observed after 48 and 72 h of exposure (Fig. 2c).

The data of Trypan Blue dye test also demonstrate that γ-Fe_2_O_3_-DMSA (60 and 80 µg/mL) is not cytotoxic to MSCs, approximately 98 % of cells remained alive after 24 h of incubation. Interestingly, this test also indicated that at least 97.6 % of the cells exposed to Au-DMSA (52 and 90 µg/mL) also survived (Fig. 2d).

**Fig. 3:**
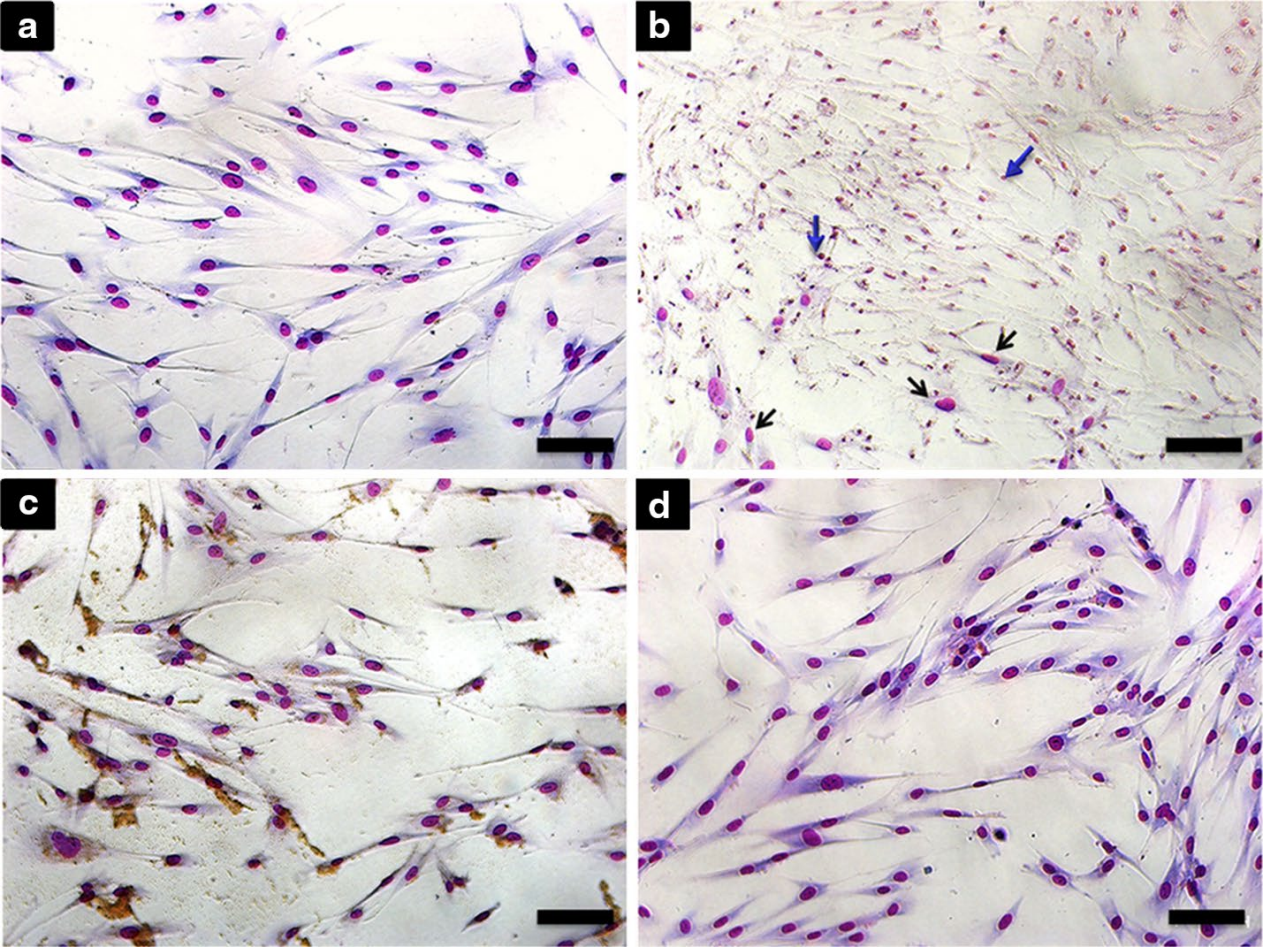
Analysis of MSC morphology by Instant Prov staining kit. **a** Negative control group. **b** Positive control group, with pyknotic nuclei (*blue* *arrows*) and normal nuclei (*black* *arrows*). **c** MSC incubated with Au-DMSA (90 μg/mL) and **d** with γ-Fe_2_O_3_-DMSA (80 μg/mL) for 24 h. *Bars* 50 micrometers (μm)

The morphological analysis of MSC under light microscope (Fig. 3) showed, as expected,that the negative control group MSC (Fig. 3a) presented a spindle form and a large nucleus. Otherwise, cells in the positive control group (Fig. 3b) showed pyknotic nuclei, a sign of apoptosis, after being cultured in serum-depleted media for 24 h. MSCs exposed to 80 µg/mL Fe_2_O_3_-DMSA (Fig. 3c) and to 90 µg/mL Au-DMSA (Fig. 3d) for 24 h were similar to the negative control MSCs, without cell shrinkage or pyknosis.

### Nanoparticle uptake

The amount of γ-Fe_2_O_3_-DMSA nanoparticles (80 μg/mL) uptaken by MSCs after 24 h, measured by Prussian Blue colorimetric quantification indicated that each cell contain approximately 17 pg of iron, while control cells only 5 pg. ICP-OES measurement indicated that each MSC retained approximately 4 pg of gold after exposure to Au-DMSA (90 μg/mL) for 24 h.

**Fig. 4:**
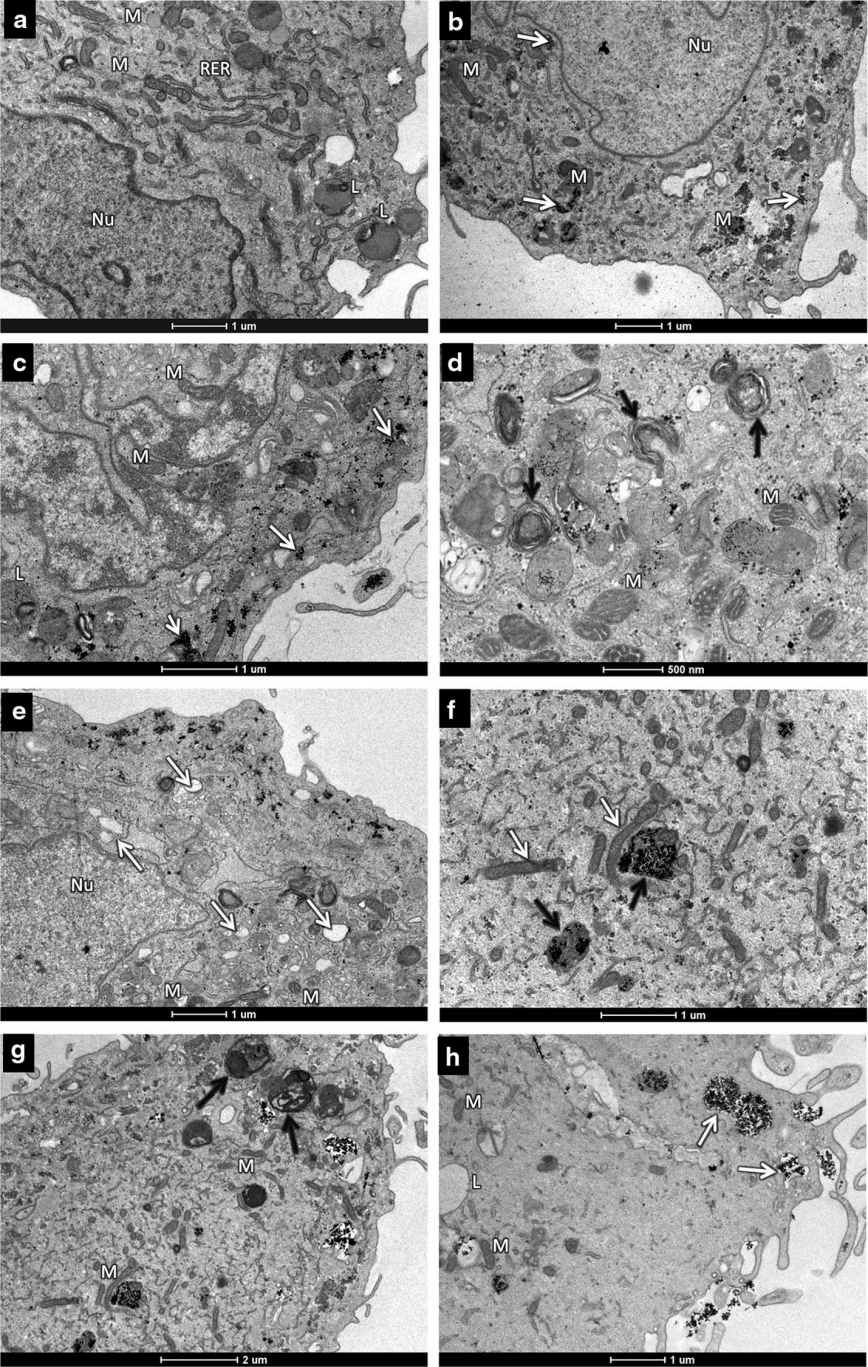
Transmission electron microscopy micrographs of labeled MSC. **a** Unlabeled MSC (control). **b** γ-Fe_2_O_3_-DMSA labeled MSC (80 µg/mL); the white arrows point some of the uptaken nanoparticles. **c** MSC labeled with Au-DMSA (90 µg/mL); the *white arrowheads* point some of the uptaken nanoparticles. **d** Cells exposed to Au-DMSA for 24 h presented myelin figures (*black arrows*) and **e** more electron-lucent structures (*white arrows*) compared to unlabeled cells. **f** In turn, after 24 h of exposure toγ-Fe_2_O_3_-DMSA, MSC presented signs of mitochondrial toxicity: mitochondria full of nanoparticles are swollen and degenerated (*black arrows*), as compared to organelles without them (*white arrows*). **g** These cells also presented some myelin figures (*black arrows*). **h** Lastly, γ-Fe_2_O_3_-DMSA nanoparticles were stored in vesicles (*white arrows*). *Nu* nucleus, *M* mitochondria, *RER* rough endoplasmic reticulum, *L* lipid

In the Fig. 3, TEM analysis confirms the uptake of nanoparticles by MSC (Fig. 4a–c). Fe_2_O_3_-DMSA (Fig. 4b) and Au-DMSA (Fig. 4c) are free in the cell cytoplasm, inside vesicles, or in different cellular compartments, particularly into mitochondria.

Compared to unlabeled MSCs, Au-DMSA labeled cells presented a similar ultrastructure, however, few differences were observed: concentric electron dense myelin figures (Fig. 4d) and electron-lucent vesicles (Fig. 4e). Likewise, γ-Fe_2_O_3_-DMSA labeled cells presented swollen and degenerated mitochondria, full of iron in their ridges (Fig. 4f) and myelin figures (Fig. 4g). Lastly, γ-Fe_2_O_3_-DMSA labeled MSC had higher amounts of nanoparticles into cytoplasmic vesicles, different to Au-DMSA labeled cells (Fig. 4g).

### Impact of DMSA-nanoparticles in MSC physiology

#### MSC differentiation

**Fig. 5:**
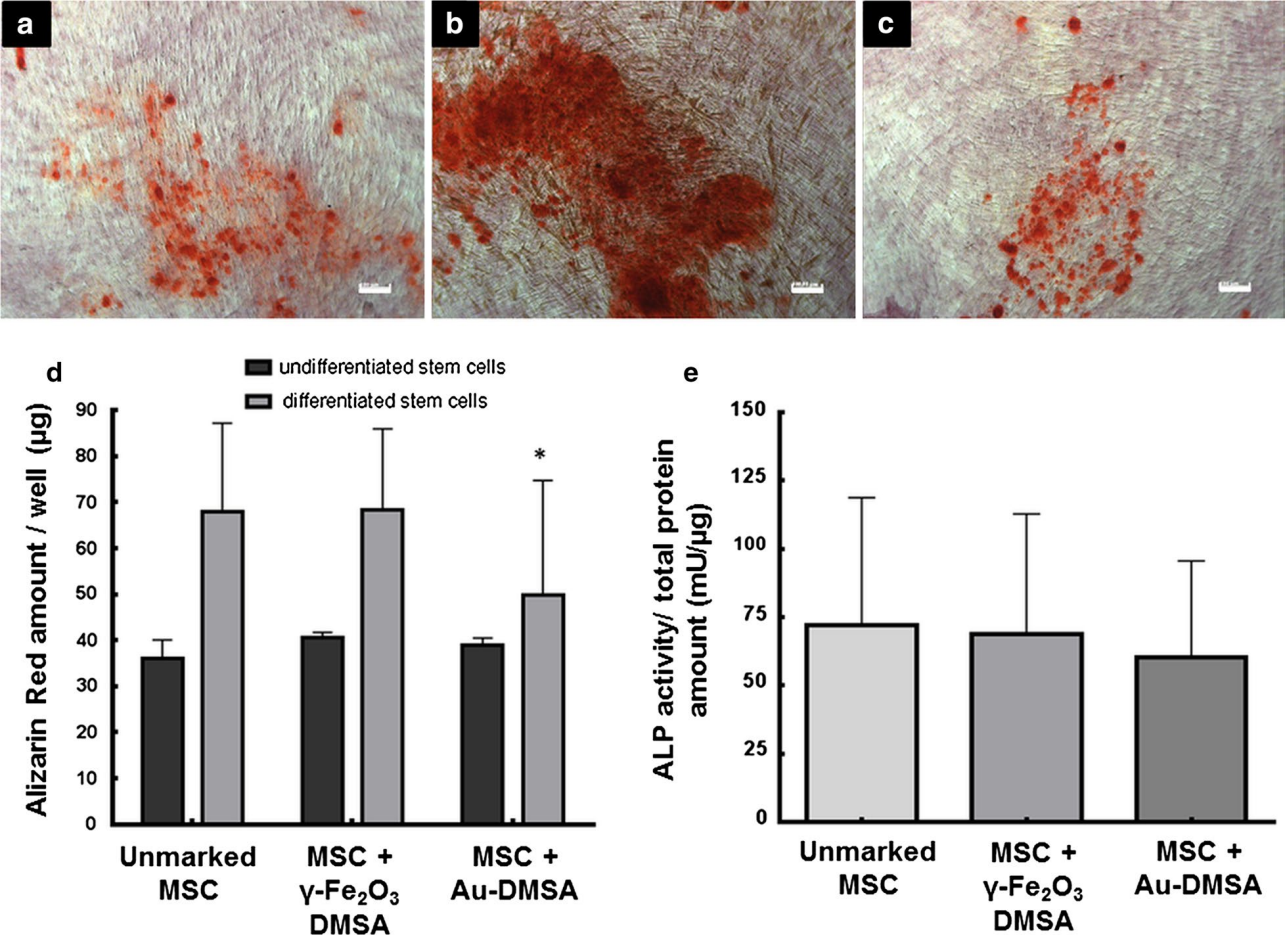
MSC osteogenic differentiation assay. **a**–**c** Cytochemical analysis of differentiated MSC monolayers in light microscopy, with Alizarin Red, in order to evidence the formation of mineralized nodules. These nodules were present in control cells (**a**), in γ-Fe_2_O_3_-DMSA labeled cells, (**b**) and in Au-DMSA labeled cells (**c**). **d** Quantification of alizarin red incorporated in monolayers of differentiated and non-differentiated MSC; (*) Significant reduction in the group “MSC + Au-DMSA” compared to the groups “Unmarked MSC” and “MSC + γ-Fe_2_O_3_-DMSA” (p < 0.05). **e** Measurement of alkaline phosphatase (ALP) activity: data are represented as the average ratio of ALP activity and total protein content, with the respective standard deviations. There was no significant difference between control and experimental groups (p > 0.05). *Bars* 100 µm

After 24 days of treatment with osteogenic medium, we observed the formation of calcified nodules in MSC monolayers of all experimental groups (Fig. 5a–c). However, it was possible to see that there were fewer calcified nodes in the cells with Au-DMSA, while there were more in MSC monolayers with γ-Fe_2_O_3_-DMSA. This difference was confirmed after the measurement of the amount of Alizarin Red (ARS) absorbed by mineralized nodules (Fig. 5d).

In order to corroborate this data, we performed the measurement of alkaline phosphatase (ALP) activity in labeled and unlabeled MSC, using* p*-nitrophenylphosphate as substrate. Contrary to what was seen in ARS measurement test, there was no statistically significant difference between control cells and both experimental groups (Fig. 5e).

**Fig. 6:**
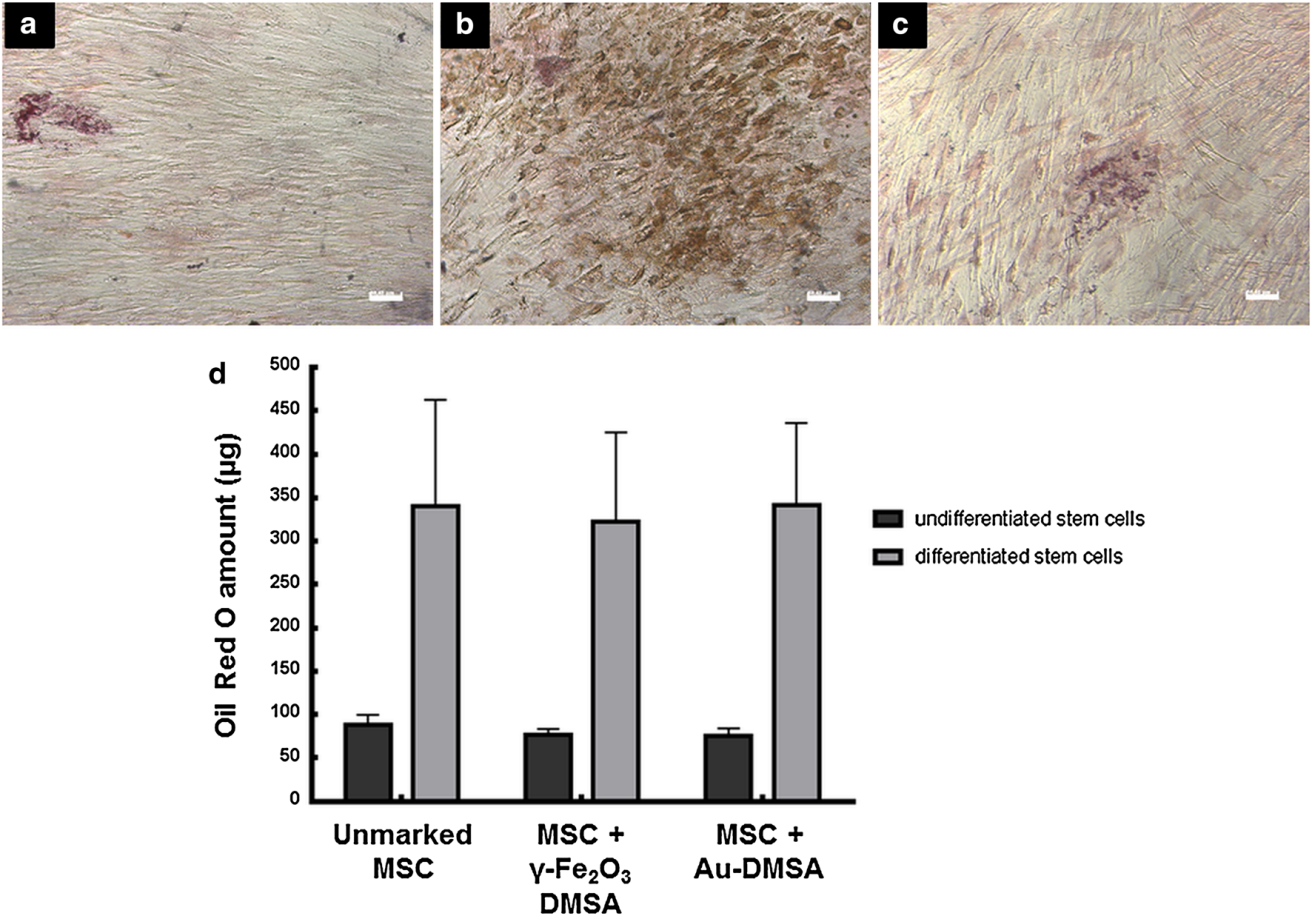
MSC adipogenic differentiation assay. **a**–**c** Oil Red O cytochemical analysis of differentiated MSC monolayers in light microscopy, with in order to evidence the formation of intracellular lipid vesicles. These vesicles were seen in control cells (**a**), in γ-Fe_2_O_3_-DMSA labeled cells, (**b**) and in Au-DMSA labeled cells (**c**). **d** Quantification of Oil Red O incorporated in monolayers of differentiated and non-differentiated MSC, with no significant difference between control and experimental groups (p > 0.05). *Bars* 50 µm

Intracellular lipid vacuoles were observed in MSCs after 24 days of incubation with adipogenic medium, showing that differentiation occurred in control and experimental groups (Fig. 6a–c). No difference in the amount of Oil Red O dye from differentiated MSC monolayers was observed in all experimental groups (Fig. 6d).

#### MSC proliferation and lymphocyte suppression

**Fig. 7:**
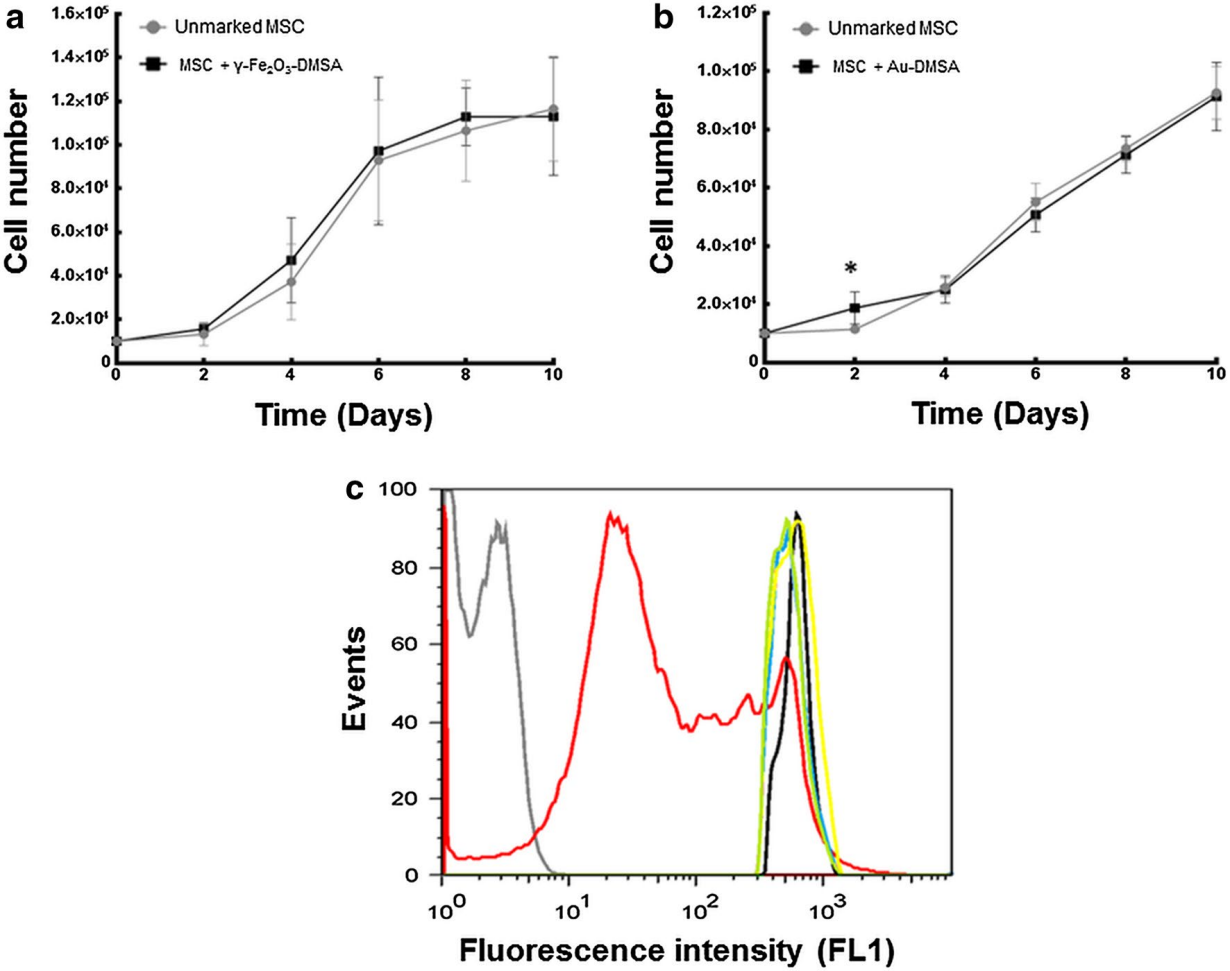
**a**, **b** MSC proliferation curves. **a** Cells were incubated for 24 h with DMEM-LG (*filled circle*), or with DMEM-LG with diluted γ-Fe_2_O_3_-DMSA (*filled square*) (80 μg/mL), then were plated and counted after different times. There was no significant difference between the experimental groups (p > 0.05) in any count times. **b** Cells were incubated for 24 h with DMEM-LG (*filled circle*), or with DMEM-LG with diluted Au-DMSA (*filled square*) (90 μg/mL), then were plated and counted after different times. (*) Significant increase in Au-DMSA labeled MSC amount compared to the control group only on the second day (p < 0.05). **c** Analysis by flow cytometry of CFSE-marked lymphocytes, co-cultured with labeled and unlabeled MSC. The spectra shown are representative of assays performed in triplicate. *Gray Line* Lymphocytes not marked with CFSE; *Black line* not activated marked lymphocytes; *Red Line* activated marked lymphocytes; *Blue line* activated lymphocytes co-cultured with MSC; *Yellow line* activated lymphocytes co-cultured with γ-Fe_2_O_3_-DMSA labeled MSC; *Green line* activated lymphocytes co-cultured with Au-DMSA labeled MSC

The results of total cell number by trypan blue staining, in order to assess the proliferative potential of labeled MSC are expressed in Fig. 7a, b. According to the individualized data analysis, there were no significant differences among the number of cells labeled with γ-Fe_2_O_3_-DMSA and control cells in any times (Fig. 7a). Au-DMSA labeled MSC showed cell number increase after 2 days of incubation (Fig. 7b).

To verify whether nanoparticle uptake influence the characteristic capability of MSC to cause unspecific lymphocyte suppression in vitro a lymphocyte proliferation test was performed. Lymphocytes were labeled with CFSE and their proliferation rates were analyzed by flow cytometry (Fig. 7c). Their proliferation leads to an intracellular reduction of the fluorescent tracer, decreasing its intensity, as shown in the Fig. 7c (red line). Because of the activation (and subsequent division) of these mononuclear cells in the control group, there are several populations with different amounts of marker, therefore, the red line span multiple fluorescence intensity values. Based on this, the representative curve of the negative control group that does not contain activated lymphocytes (black line), remained narrow. Finally, it was found in this experiment that lymphocytes, either after the co-cultivation with unmarked MSC (blue line), either with γ-Fe_2_O_3_-DMSA labeled MSC (green line) or Au-DMSA marked cells (yellow line), not proliferated.

Thus, all these results suggest that exposure to γ-Fe_2_O_3_-DMSA and Au-DMSA nanoparticles, at the tested concentrations, does not cause toxic effects to MSC and do not change their physiology. Therefore, both inorganic nanoparticles are biocompatible with MSCs.

#### Computed tomography analysis

**Fig. 8:**
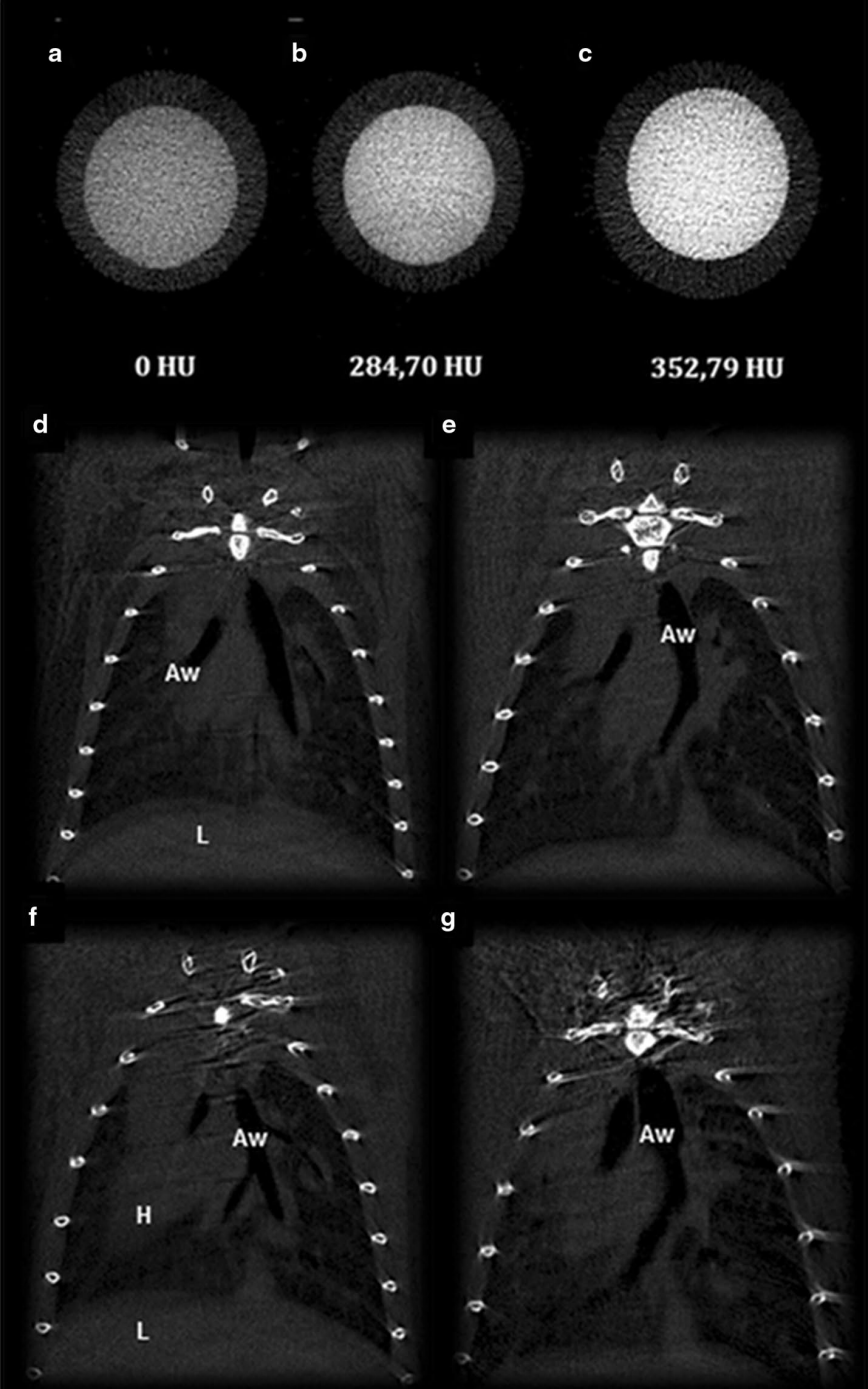
Au-DMSA potential as a tracer for MSC tracking in computed microtomography. **a**–**c** Analysis of MSC precipitates in Sky-Scan 1640 microtomograph in which cross-sections of samples in Eppendorf tubes are represented: **a** water, **b** unlabeled MSC, **c** MSC labeled with Au-DMSA. **d**–**g** Sky-Scan 1640 images of longitudinal sections of mice. The animals were immediately analyzed after intranasal instillation with unlabeled cells (**d**) or with Au-DMSA labeled cells (**e**). Five days later, animals instilled with unlabeled cells (**f**) and Au-DMSA labeled cells (**g**) were analyzed again. *Aw* airways; *H* heart; *L* liver

Firstly, a test was performed in which tubes containing precipitates of unlabeled MSC or Au-DMSA labeled cells (90 μg/mL, 24 h) were scanned in a micro-CT equipment to assess whether nanoparticles generate adequate contrast (Fig. 8a–c). The parameters of acquisition and image reconstruction were adjusted similarly to those used in analysis with mice. A tube containing water served as control, and the signal generated by the liquid was then considered as 0 (zero) Hounsfield (HU). The values in Hounsfield scale for each sample were: 284.70 HU in control MSC and 352.79 HU in Au-DMSA labeled MSC.

Although the Au-DMSA has generated a visible contrast in images of cell precipitates, labeled MSCs were not detected by the device after its inoculation in mice in any analyzed times (Fig. 8d–g).

#### MSC magnetic targeting

**Fig. 9:**
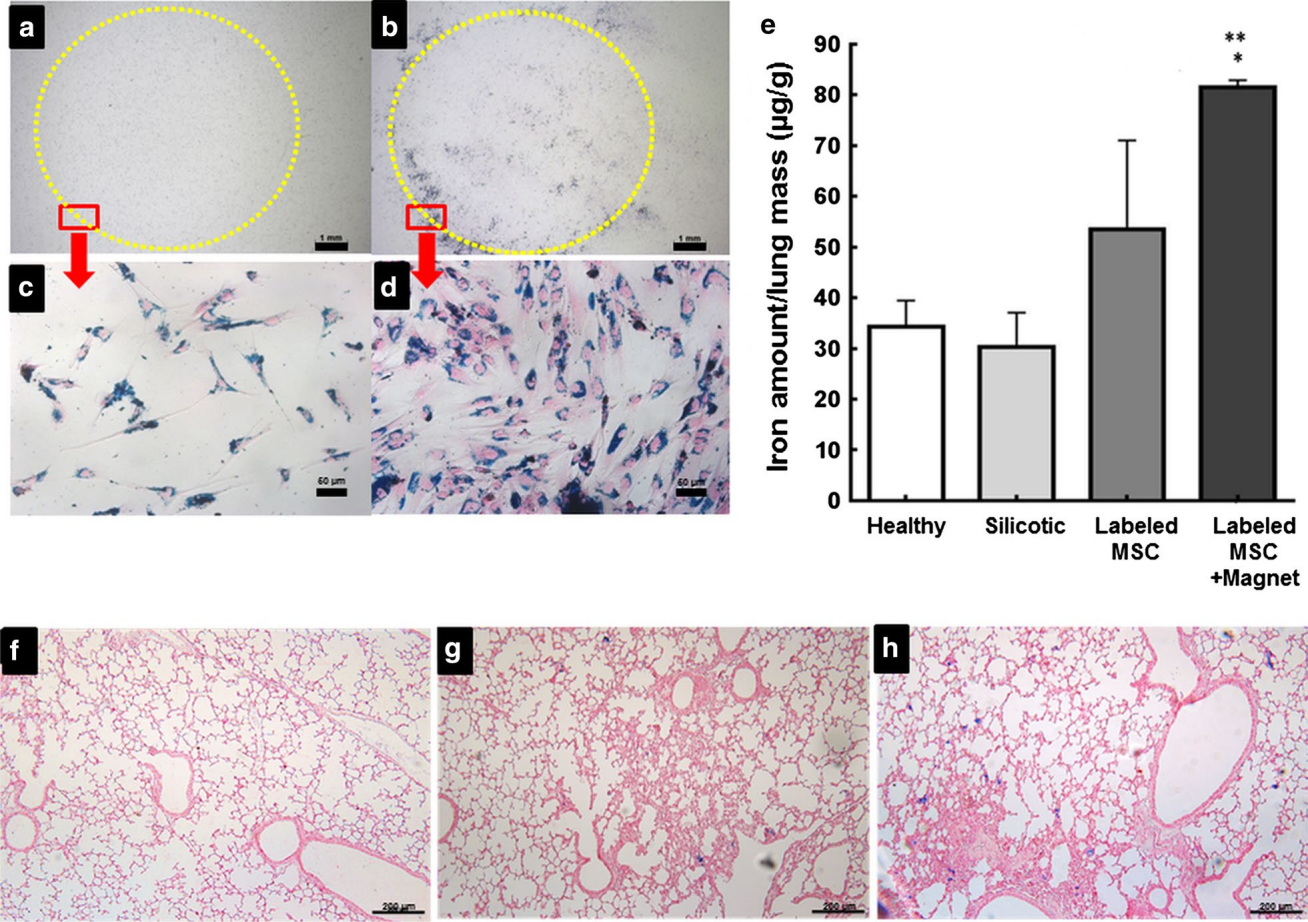
γ-Fe_2_O_3_-DMSA as a potential agent for MSC magnetic targeting. **a**–**d** Magnetic responsiveness test in vitro. **a**, **b** γ-Fe_2_O_3_-DMSA labeled cells were seeded in culture plates with a fixed plastic piece (**a**) or a circular magnet (**b**). The region in *orange* corresponds to the site where the materials were fixed. **c**, **d** The culture plates were examined by light microscopy in order to demonstrate the difference in amount of cells present in regions *highlighted in red*. **e** Iron measurement by colorimetric dosage of Prussian blue. Data refer to the mean ± SD from the mass of iron present in lungs, divided by lung’s weight. **f**–**h** Histological analysis of MSC retention in silicotic mice lungs. The slides were stained with Prussian Blue technique and contrasted with neutral red, evidencing iron from the γ-Fe_2_O_3_-DMSA in blue. **f** Healthy animal treated with saline **g** Animals treated with γ-Fe_2_O_3_-DMSA labeled MSC without external magnets **h** Animal treated with labeled MSC and with external magnets

In order to verify if γ-Fe_2_O_3_-DMSA labeled MSC (80 µg/mL, 24 h) became magnetically responsive, a test was previously performed in vitro (Fig. 9a–d). Labeled cells were maintained in culture in the presence of an external circular magnet or of a similar size plastic (control). It can be seen that the cells migrated toward the edge of the circular magnet (Fig. 9b, d), unlike the control group cells (Fig. 9a, c).

Next, we test the potential of in vivo magnetic targeting of γ-Fe_2_O_3_-DMSA labeled MSCs in an experimental mice model of lung silicosis. Histological analysis of experimental silicosis mice inoculated with γ-Fe_2_O_3_-DMSA labeled MSC and with magnets fixed in their thoracic region presented higher iron content in their lung tissue than animals that did not hold external magnets (Fig. 9e). The light microscopy images also corroborate this data (Fig. 9f–h): animals treated with marked cells and holding magnets had more blue spots, corresponding to γ-Fe_2_O_3_-DMSA, compared to animals without magnets.

## Discussion

This work had two main purposes: (1) to evaluate biocompatibility between MSC and DMSA-coated inorganic nanoparticles, verifying cytotoxic effects or physiological alterations; and (2) to test Au-DMSA and γ-Fe_2_O_3_-DMSA as agents for MSC in vivo tracking and for MSC magnetic targeting, respectively. Firstly, our results demonstrated the absence of toxic effects on MSC and suggested no significant changes in physiological parameters such as cell differentiation, proliferation and immunomodulation, at the concentrations tested. In addition, Au-DMSA nanoparticles had a poor performance as MSC tracers when analyzed on a microtomograph; while γ-Fe_2_O_3_-DMSA showed to be good agent for magnetic targeting.

The results of MTT and trypan blue analysis demonstrated the low toxicity of γ-Fe_2_O_3_-DMSA on MSC, corroborating with other studies [34, 35, 47, 48]. Although iron oxide catalyze free radicals production, chemical surface modifications make them safer materials for biological applications [34, 49, 50]. For example, Auffan et al. [34] suggested that is difficult to remove DMSA coating from the nanostructure (different than dextran or albumin), preventing direct contact of the cells with iron, protecting them from possible toxic effects. Thus, if the nanoparticle coating is easily degraded, the core can then react with cellular biomolecules [49].

Moreover, Chen et al. [47] demonstrated that IONPs oxidative activity depends on the acidity of intracellular microenvironment in which they are located. In lysosomes (low pH), IONPs produce more hydroxyl radicals (OH^−^), increasing cell damage induced by H_2_O_2_. In neutral environments, IONPs break H_2_O_2_ into H_2_O and O_2_ [47]. In our work, TEM images showed few γ-Fe_2_O_3_-DMSA in structures similar to lysosomes; most was located in MSC mitochondria, which are neutral pH organelles. Considering that, γ-Fe_2_O_3_-DMSA could not exert toxic effects to receptor MSC.

Otherwise, MTT tests showed significant differences between unlabeled MSC and Au-DMSA labeled MSC, suggesting harmful effects in their mitochondria. In spite of this, our data followed the same pattern found by Fan et al. in 2009, which studied biocompatibility between gold nanoparticles (Au-NP) and MSC, using slightly lower gold concentrations (71,1 µg/mL) [51]. This similarity suggests that MSC have a natural sensitivity to Au-NP, that is, the toxic effects observed are not related to Au-DMSA features, but to MSC cellular mechanisms that make them more or less vulnerable to label [50]. In addition, Fig. 1c shows that MSC viability increases 48 and 72 h after exposure to Au-DMSA, which illustrates *cellular recovery* described by Mironava et al. [24, 26]: damage caused by Au-NP marking are not permanent because, after nanoparticle exposure, gold cytoplasmic levels diminish and cells can completely recover their structures and/or altered functions. Interestingly, despite deleterious effects on mitochondrial metabolism (Fig. 2b), trypan blue tests (Fig. 2d) and cell morphology analysis (Fig. 3) suggested that the Au-DMSA did not cause MSC death 24 h after exposure. So, we had to verify if these mitochondrial damages led to changes in important physiological parameters of cells; what was accomplished in the following experiments.

Some reports suggest that nanoparticles actively interact with plasma membrane receptors, modulating signal transduction pathways, and inducing proliferation, immunomodulation, apoptosis or differentiation [52]. These harmful effects caused by altered cellular communication pathways cannot be detected only with viability tests, such as MTT and Trypan Blue. Therefore, MSC differentiation, MSC proliferation and lymphocyte suppression tests were also performed. It is important to note that our study was the first that investigated nanoparticles effects on MSC immunomodulatory capacity. At the concentrations tested, both γ-Fe_2_O_3_-DMSA and Au-DMSA did not change this intrinsic property of cells, essential for the success of cellular therapies.

In differentiation tests, MSC incubated with γ-Fe_2_O_3_-DMSA showed no changes in adipogenesis and osteogenesis capacity, confirming previously published work [27]. Unlikely, Au-DMSA reduced MSC osteogenesis, corroborating Fan et al. data [51]. Although many studies in the literature describe Au-NP stimulus on osteogenic differentiation and mineralization [29, 53, 54], Fan et al. verified a reduction in ALP activity and in calcium deposition, similar to our findings (Fig. 4). This disagreement may be caused by the Au-NP concentration used to label MSC: while 1.97 × 10^−4^ µg/mL induced differentiation in Zhang et al. report [54], 71.1 µg/mL inhibited osteogenesis in Fan et al. study [51]. The higher Au-NP amount caused cytotoxic effects by oxidative stress, which represses both osteogenic and adipogenic differentiation [51].

In proliferation assays, labeled MSC proliferation rates presented no differences compared to respective control groups, except a significant increase in Au-DMSA labeled MSC amount, on the 2nd day. This result disagrees with data presented by Mironava et al. [26]: MSC were incubated for 72 h with Au-NP (45 nm; 13, 20 and 26 µg/mL) and proliferation rates were lower than controls in all samples. The most notable difference between our study and Mironava et al. study was the incubation times—MSC were exposed to Au-NP during 24 and 72 h, respectively. Importantly, Mironava et al. aimed to observe long-term effects (3–6 days) and *cellular recovery* on different cell lines exposed to Au-NP, highlighting the cytotoxic effects over long incubation times. On the other hand, to date, there are no reports of stimulation of MSC proliferation by Au-NP. Together, all these data indicate that Au-DMSA nanoparticles are innocuous labels for MSC.

Some reports have described that Au-NPs enable cell tracking in vivo by using computed tomography [5, 17]; however in our study we could not detect the signals generated by the Au-DMSA in a microtomograph. Some facts may explain this disagreement: (1) It is possible Au-DMSA provided contrast to MSC, but not enough to distinguish them from the connective tissue associated with bronchi or the fibrous own tissue; (2) In our study, we injected an amount of labeled MSC lower than that used in other studies, for example, Menk et al. [17] inoculated 10^7^ cells , while we used between 0.5 and 1 × 10^6^ cells, an amount we considered safe to prevent lung occlusions; (3) The different tomography equipments used in our study and Menk et al. [17] study may also be a cause for the discrepancy observed. Menk et al. used an equipment connected to a source of synchrotron radiation, resulting in images with higher contrast and better visibility of details such as cells labeled with Au-NPs [55]. In addition, Au-NPs-labeled MSC are usually inoculated systemically in tracking studies [5, 17]. However, in this work, we decided to check the feasibility of inoculating MSC via intranasal route which is a more direct route for respiratory disease treatments. On the other hand, our in vitro and in vivo assays demonstrated γ-Fe_2_O_3_-DMSA potential for magnetic targeting of MSC. Qualitative (histological sections analysis) and quantitative assays (Prussian blue colorimetric dosage) showed a higher amount of iron in mice lungs which had magnets fixed in their bodies, suggesting a greater MSC retention. This was the first study that explored magnetic targeting of cells to the lung and our results raise the possibility of using this technique to enhance the effects of cellular therapy in lung diseases such as silicosis. Although it is known that pulmonary capillaries retains much of the systemically infused cells, there is evidence that this retention is not permanent [56], which may explain why beneficial effects of these cells in models of lung diseases do not last.

The findings of this study showed that iron and gold nanoparticles functionalized with DMSA, in the tested concentrations, were effectively uptaked by MSC, did not exert toxic effects, and did not induced changes in MSCs function (differentiation capacity, proliferation and inhibition of T lymphocytes). In addition, our results suggest the use of γ-Fe_2_O_3_-DMSA as agents for magnetic targeting of MSCs.

## Abbreviations

ALP: alkaline phosphatase
Au-DMSA: meso-2,3-dimercaptosuccinic acid coated gold nanoparticles
CFSE: carboxyfluorescein succinimidyl ester
DLS: dynamic light scattering
DMEM-LG: low-glucose Dulbecco’s modified Eagle medium
DMSA: meso-2,3-dimercaptosuccinic acid
ICP-OES: inductively coupled plasma optical emission spectrometry
IONPs: iron oxide nanoparticles
MSCs: mesenchymal stem cells
MTT: 3-[4,5-dimethylthiazol-2-yl]-2,5-diphenyltetrazolium bromide
TEM: transmission electron microscopy
XRD: X-ray powder diffraction
γ-Fe_2_O_3_-DMSA: meso-2,3-dimercaptosuccinic acid coated maghemite nanoparticles
